# Effectiveness of a Patient-Centered Dietary Educational Intervention

**DOI:** 10.31486/toj.21.0075

**Published:** 2022

**Authors:** Tiffany Wesley Ardoin, Diana Hamer, Nia Mason, Addy Reine, Lindsay Barleycorn, Diane Francis, Angela Johnson

**Affiliations:** ^1^ Department of Internal Medicine, Louisiana State University Health Sciences Center–Baton Rouge, Baton Rouge, LA; ^2^ Department of Research, Our Lady of the Lake Regional Medical Center, Baton Rouge, LA; ^3^ Manship School of Mass Communication, Louisiana State University, Baton Rouge, LA; ^4^ Department of Diabetes and Nutrition, Our Lady of the Lake Regional Medical Center, Baton Rouge, LA

**Keywords:** *Diet–healthy*, *patient education*, *video recording*

## Abstract

**Background:** A healthy diet is an important component of preventive medicine. With the changing landscape of medicine, physicians are encountering more challenges in educating patients about a healthy diet, so finding innovative ways to educate patients is imperative. This study investigated the effectiveness of an innovative educational intervention based on the United States Department of Agriculture–recommended MyPlate diet.

**Methods:** Based on the assessed need for dietary education, patients were exposed to an educational video and received a handout on the MyPlate diet. The educational video was created to be culturally relevant with patient-informed edits. The handout was taken from www.ChooseMyPlate.gov. The patients who received the intervention were compared to those who were not exposed to the intervention. Data were collected in a primary care clinic for an underserved population in fall 2018 and analyzed in spring 2019 through patient-completed surveys and physician reporting on patient interactions. Data were analyzed using descriptive statistics, *t* tests, chi-squared models, and repeated measures analysis of variance.

**Results:** Among 320 patients, 169 patients were exposed to the educational intervention. Intervention patients had better knowledge of the MyPlate diet (*P*=0.009), felt it would be easier to change their diet (*P*=0.03), and were more motivated to have conversations about diet with their physician (*P*=0.04) compared to those who were not exposed. Patients also enjoyed the video overall.

**Conclusion:** This study shows that using multiple modalities including a patient-centered video and handouts to educate patients about diet is enjoyable to patients and effective in teaching, motivating change, and encouraging communication between patients and physicians.

## INTRODUCTION

A healthy diet is the cornerstone of preventive medicine. The 2018 US Burden of Disease Report showed that poor diet is one of the leading causes of death in the United States.^1^ A comparative risk assessment model revealed that suboptimal intake of healthy foods such as fruits and vegetables and excessive intake of less healthy foods such as processed meat and excess sugars was associated with 45% of all cardiometabolic deaths.^2^ Furthermore, an abundance of evidence shows the association of a healthy diet with improved health outcomes. For example, the Mediterranean diet alone has been shown to have a relative risk reduction of 30% in primary cardiovascular events.^3^ This evidence showing that healthy eating improves health outcomes demonstrates that dietary counseling is an imperative component of patient education and preventive medicine.

Patient education has been proven to improve health outcomes if done effectively.^4^ However, with increasing health care demands, physicians have limited time to educate their patients. Physicians spend 27% of their time with patients and spend the remaining time documenting in the electronic medical record and doing clerical work.^5^ Moreover, the lack of nutrition education provided in medical schools further impacts patient education on a healthy diet. Only 27% of US medical schools include the recommended 25 hours of dietary education, and most of this time is based in biochemistry.^6^

Several studies have attempted to incorporate dietary education into clinical settings, but the designs have limited applicability. For example, one study protocol used wearable devices to monitor exercise, food diaries, and intensive nutrition therapy from registered dietitians.^7^ Based on the literature to date and the need for better dietary education in our clinics, we created a translatable patient educational intervention to teach our patient population about a healthy diet. This intervention included a MyPlate diet handout from the United States Department of Agriculture (USDA) and a video created in partnership with our undergraduate marketing department with health literacy and cultural relevancy in mind. Creating a dietary video with special attention to literacy level and Louisiana culture, in addition to allowing patients to inform the final edits to the video, made for a truly patient-centered educational intervention.

## METHODS

### Needs Assessment

In our institution's primary care and subspecialty care clinics, we assessed the need for patient education regarding a healthy diet and other potential educational topics by implementing a comprehensive needs assessment survey (Appendix A) in fall 2016. This survey incorporated multiple validated surveys, including the Food Consumption and Accessibility Scale,^8^ the Medication Understanding and Use Self-Efficacy Scale,^9^ the Culig Adherence Scale,^10^ the Exercise Benefits/Barriers Scale,^11^ and the 12-item Short Form Health Survey, version 2.0 (SF-12v2).^12^

During a 4-month period, 176 patients completed the surveys. The majority were female, African American, and recipients of the Louisiana Supplemental Nutrition Assistance Program (SNAP). Overall, surveyed patients scored below the population average in the physical component and mental component of the SF-12v2. The standard average score for the physical and mental component questions is 50. However, our patients’ average scores ranged between 38.26 and 39.54 on the 5 physical component questions and between 40.77 and 45.98 on the 5 mental component questions, with 76% and 47% of patients scoring below the national average, respectively. This finding was expected given that in our clinic population, 51% of patients are classified as obese. Also, our population is underserved, with multiple barriers to health care and with 60% of patients on Medicaid and 10% uninsured. However, the most notable result was the lack of reliability with the food consumption and food accessibility portions of the survey. The Cronbach alpha measuring the internal consistency of the previously validated Food Consumption and Accessibility Scale was only α=0.371, suggesting that our patients did not understand or adhere to healthy diet behaviors consistently.

This information, coupled with the high percentage of patients who receive SNAP, led us to focus our efforts on providing a patient educational intervention based on the USDA-recommended MyPlate diet. We chose the MyPlate diet because it is comprehensive yet easy to understand, and the information could be presented in a culturally relevant way to our patients.

### Intervention Development

After determining the need for dietary education in our clinics, we performed a literature search to find the most effective and innovative ways to teach patients. Because of limited time during clinic visits, the use of technology to improve patient education has been a growing area of research interest. Studies have shown that verbal discussions alone are the least effective form of patient education, while technology has been shown to improve knowledge, lessen anxiety, and improve satisfaction with patient-centered health education.^13^ Furthermore, using multiple media modalities has been shown to be more effective than using a single modality.^13^ We decided to use a combined video/handout educational intervention to teach patients about a healthy diet and attempt to impact health behaviors and health outcomes.

**Figure 1.**
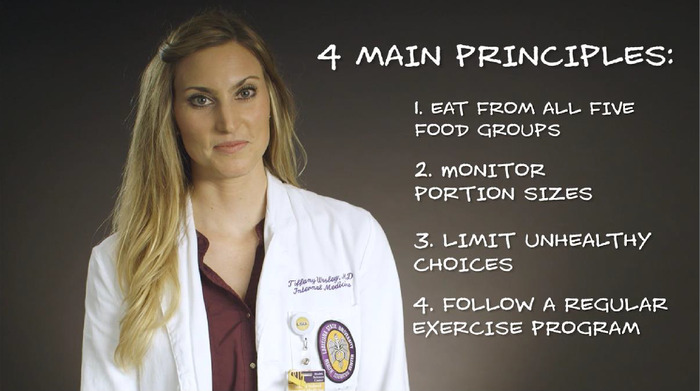
Screenshot of video portraying simple messaging with recognizable, friendly clinic physician.

Because a commercially available MyPlate video tailored to the needs of our patients was not available, we created a patient-centered, culturally relevant, 6-minute dietary video based on the MyPlate dietary guidelines. Collaborative meetings with physicians and a mass communication professor were held during summer 2017 to discuss a video that would create an engaging and welcoming message for patients and encompass the major pillars of the MyPlate diet in a fun and easy-to-understand manner. We designed the preliminary script after studying the diet in detail and aligned the script with verbiage similar to how physicians traditionally counsel patients in clinic. A registered dietitian helped to edit the script for accuracy regarding the diet. The script was written below an 8th grade level of education, and cartoon graphics were used throughout the video to increase appeal among viewers. To create the video, we partnered with a local media company. By using a local physician as the video narrator, we aimed to give patients a feeling that the dietary recommendations were made specifically for them by a physician in the clinic. Also, we felt that having a local physician in the video would be best for patient comfort, education, and adherence (Figure 1).

**Figure 2.**
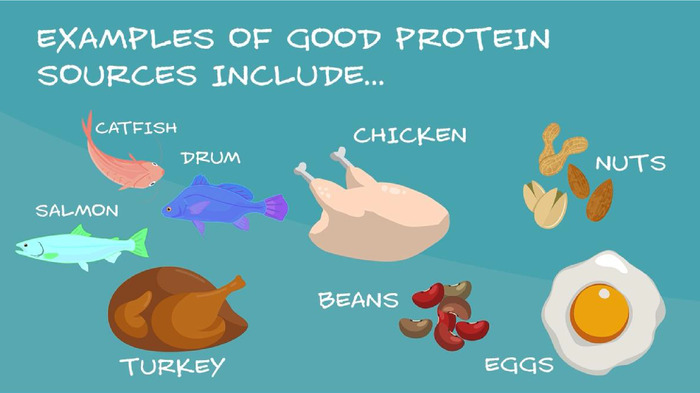
Screenshot of video demonstrating the simple messaging used and some of the changes informed by patient feedback, including showing multiple examples of fish.

A patient focus group provided feedback on the pilot video in December 2017. Nine participants from a population similar to our clinic patients attended the focus group meeting at a local library where they were divided into 2 groups. Each group watched the video twice and then participated in discussions with 2 of the investigators. Overall, the feedback was positive; participants indicated that the video was enjoyable and easy to understand. Six themes emerged from the thematic analysis: low MyPlate knowledge and awareness, some unhealthy food habits and beliefs, desire to learn more, great excitement and inquisitiveness about the video, perception of the video as effective, and the need for more food choice options. Participants expressed the opinion that such a tool would make a difference in the clinics. The participants provided additional constructive feedback to improve the video before the launch of the educational intervention. Their feedback informed the final edits to the MyPlate video that included providing examples of different types of fish and ensuring the message included using the knowledge learned in the video to teach the whole family about a healthy diet (Figure 2).

The final video was 6 minutes in length and approved by the sponsoring institution. The handout used in conjunction with the video was taken from the MyPlate website (Appendix B).^14^

### Study Design and Participants

The educational intervention consisted of playing the video in the patient room while patients waited for their physicians and providing the MyPlate handout for patients to take home. Clinical staff were educated about the diet and intervention so they could answer any questions patients had during their visit.

This quasi-experimental postintervention study included patients from 1 location of our institution's primary care and subspecialty care clinics. Patients were recruited during clinic visits in fall 2018. Patients had to be at least 18 years or older, English speaking, and able to read and grant consent. The Louisiana State University Health Sciences Center Institutional Review Board approved the study by expedited review.

### Intervention, Survey, and Medical Record Documentation

One group was exposed to the educational intervention (video and handout) during a 9-month period in the primary care clinic, and the other group was not exposed as they were seen in the subspecialty care clinics where the intervention did not take place. A small proportion of patients had appointments in both primary care and subspecialty care clinics during the study period.

Six months after the launch of the intervention, we conducted surveys in both clinics to assess knowledge of the MyPlate diet, likeability of the MyPlate intervention, motivation to change, and the likelihood of communicating with their physicians about a healthy diet (Appendix C). No baseline number of clinic visits was required to take the survey, and patients in both the intervention group and nonintervention group were recruited in the same manner. Both groups were informed that the survey was administered to gather information about familiarity with the MyPlate diet, knowledge of dietary recommendations, and opinions about personal diet habits. The survey was provided to patients before they were directed to a patient room, and they were able to drop the completed survey into a box once they were in the patient room.

Neither group was informed that they would be seeing an educational video or receiving a handout. However, the intervention group would have been exposed to the video and received the handout to take home at the end of a previous visit. Patients who had seen the video during previous visits were able to disclose so on the survey. The time between seeing the video and taking the survey varied based on the timing of follow-up visits, and the timing was not standardized to improve feasibility of the assessment. This sample design was intended to reach all adults in our primary care clinic who could potentially benefit from our nutrition-based patient educational intervention. All patients recruited followed through with the intervention.

We used the first 30 anonymous surveys that were completed to test the validity and reliability of each question. The validation of this survey was complete after we established face validity, interpreted the results of the first 30 surveys to ensure the answers appropriately reflected the questions asked, and determined internal consistency. We included the first 30 surveys in the analysis because minimal changes were made to the survey.

As part of the assessment of this intervention, resident physicians used a dotphrase (smartphrase with prepopulated questions and dropdown answers) in the electronic medical record to document discussions about diets with their patients. The dotphrase was incorporated into resident note templates and used for both primary care visits and subspecialty care visits during the 9-month intervention. The dotphrase included the following yes/no questions: (1) Was there a conversation about diet during the clinic visit today? (2) Did the patient initiate the conversation about diet? (3) Did the patient ask specifics about the MyPlate diet video? These data were collected in fall 2018.

### Outcomes

The primary outcome was a higher level of content knowledge regarding the MyPlate diet based on scores from the Understanding Content section of the survey. Secondary outcomes included increased thoughts about adhering to a healthy diet, motivation to improve diet, and increased discussions with physicians regarding the MyPlate diet. These outcomes were measured through survey questions and the dotphrase records documenting communication about diet between patients and physicians.

### Statistical Analysis

For the interpretation of the survey, we described the baseline demographics of our cohort including sex, race, educational level, and age using chi-squared tests (or *t* test for age) to discern any significant differences between groups. We used descriptive statistics to interpret the video and handout likeability survey questions based on Likert scale mean values. We assessed the prevalence of patients in subspecialty care vs primary care and their exposure to the educational intervention using chi-squared tests. We performed *t* tests independently on patient content knowledge of the MyPlate diet, motivation to alter diet, and likelihood to discuss diet with physicians, with viewing the MyPlate instructional video and viewing the MyPlate handout as independent variables. A repeated measures analysis of variance design was used to interpret the dotphrase results.

## RESULTS

We had 320 patients complete the survey; 169 (52.8%) of these patients were exposed to the intervention and 151 (47.2%) were not. Baseline characteristics of the study population are described in Table 1. Overall, the study population was reflective of our clinic characteristics. The population was 67% to 69% female and 63% to 67% Black. The average patient age was 49 years, and patients had a wide diversity of educational levels. Although the difference between groups was not statistically significant, we noted a trend toward higher education in the educational intervention group.

**Table 1.** Patient Demographics

Variable	Educational Intervention Group, n=169	No Educational Intervention Group, n=151	*P* Value
Sex	n=160	n=142	0.8^a^
Male	50 (31)	47 (33)	
Female	110 (69)	95 (67)	
Race	n=156	n=136	0.7^a^
White	46 (29)	44 (32)	
Black/African American	104 (67)	85 (63)	
Other	6 (4)	7 (5)	
Education level	n=149	n=126	0.08^a^
Some high school	16 (11)	22 (17)	
High school/GED	45 (30)	49 (39)	
Some college	38 (26)	20 (16)	
Vocational/technical	24 (16)	18 (14)	
College degree	22 (15)	11 (9)	
Master's degree or higher	4 (3)	6 (5)	
Age, years, mean ± SD^b^	49 ± 13.18	50 ± 12.89	0.4^c^

Note: Data are presented as n (%) unless otherwise indicated.

^a^Chi-squared test.

^b^n=158 for the intervention group, and n=134 for the nonintervention group.

^c^*t* test.

GED, General Educational Development Test.

Overall, patients who saw the video and received the handout indicated that they enjoyed/liked both (Table 2). Patients agreed that the video was enjoyable, with a mean score of 3.9 (3 meaning “neutral” and 4 meaning “somewhat agree”). Patients indicated that they learned from the video (the median score was consistent with “strongly agree”) and that they would follow recommendations from the video. Regarding the handout, patients liked the handout and referred to the handout which sparked a motivation to change their dietary habits (mean scores between “neutral” and “somewhat agree”). Of note, only 18% of patients who received the handout went to the MyPlate website address listed on the handout.

**Table 2.** Perceptions of Patients Who Viewed the Video and Received the Handout, n=169

Question	Mean	Median
Enjoyed the video	3.9	4
Learned from the video	4.0	5
Will follow recommendations in the video	3.8	4
Liked the handout	3.9	4
Referred to the handout	3.4	3
Felt motivation to change after intervention	3.9	4
Went to MyPlate website, n (%)	29 (18)^a^	

Note: Mean and median responses are based on the following Likert scale: 1=strongly disagree, 2=somewhat disagree, 3=neutral, 4=somewhat agree, 5=strongly agree.

^a^165 valid answers.

Table 3 shows the differences in clinic visits, dietary content understanding, motivation to make dietary change, and patient engagement and perception between the 2 groups. When looking at the breakdown of patients exposed to the educational intervention, 92% of the exposed group was in the primary care clinic. However, we saw more crossover between clinics than expected, with 30% (n=45) of patients unexposed in the primary care clinic and 8% (n=14) of patients exposed in the subspecialty care clinics. Therefore, the 45 patients unexposed in the primary care clinic were not provided the video/handout intervention as intended, and the 14 patients in the subspecialty care clinics who were exposed must have had a recent primary care clinic appointment where they were exposed to the intervention. Regarding understanding content, the group who saw the video and received the handout had a higher mean percentage of correct questions regarding the MyPlate diet compared to the group who did not see the video and receive the handout. Although the score was only mildly better (62% correct vs 55% correct), the difference was statistically significant (*P*=0.009). When assessing patients’ motivation to change their dietary habits, although not statistically significant, patients who were exposed to the intervention trended toward having more thoughts about their diet on repeat visits and feeling more strongly that changing their diet would improve their health. Moreover, patients who saw the video and received the handout indicated it would be easier to change their diet compared to patients who did not receive the intervention, and the difference was statistically significant. The patients exposed to the intervention were also more likely to ask their doctor about diet (*P*=0.04) compared to the unexposed group. The groups reported no difference in perceived wait times.

**Table 3.** Dietary Understanding, Motivation to Change Diet, and Patient Engagement and Perception by Group

Variable	Educational Intervention Group, n=169	No Educational Intervention Group, n=151	*P* Value
Clinic			0.000^a^
Primary care clinic, n (%)	155 (92)	45 (30)	
Subspecialty care clinics, n (%)	14 (8)	106 (70)	
Dietary understanding			
Percentage of correct answers on a 7-question quiz	62	55	0.009^b^
Motivation to change diet, mean^b^			
Thoughts about diet before last clinic visit^c^	3.4	3.2	0.1
Thoughts about diet after last clinic visit^c^	3.6	3.2	0.1
Change in thoughts about diet^c^	0.2	0	0.1
Changing diet would improve health^d^	4.3	4	0.07
It would be easy to make changes to diet^d^	3.5	3.1	0.03
Patient engagement and perception, mean			
I will ask doctor about diet^d^	3.6	3.3	0.04^b^
I had to wait a long time today^d^	2.5	2.8	0.17^b^

^a^Chi-squared test.

^b^*t* test.

^c^Responses are based on the following Likert scale: 1=never, 2=rarely, 3=occasionally, 4=regularly, 5=often.

^d^Responses are based on the following Likert scale: 1=strongly disagree, 2=somewhat disagree, 3=neutral, 4=somewhat agree, 5=strongly agree.

The results of the dotphrase documentation resident physicians incorporated into their clinic notes are listed in Table 4. The residents incorporated this dotphrase more frequently during encounters in the primary care clinic where the intervention took place, with 501 dotphrase documentations in the primary care setting and only 62 in the subspecialty care setting. The patients in the primary care clinic, which comprised 92% of those exposed to the intervention (Table 3), were more likely to have a conversation about diet, were more likely to initiate the conversation about diet, and were more likely to ask specifics about the MyPlate diet.

**Table 4.** MyPlate Dotphrase Documentation, n=563

Documentation in Electronic Medical Record	Primary Care Clinic	Subspecialty Care Clinics	*P* Value
Number of dotphrase documentations	501 (89)	62 (11)	–
Conversation about diet	240 (48)	24 (39)	0.16^a^
Patient initiated conversation	95 (19)	6 (10)	0.08^a^
Patient asked specifics about MyPlate diet video	30 (6)	1 (2)	0.13^a^

Note: Data are presented as n (%).

^a^Repeated measures analysis of variance.

## DISCUSSION

In this cohort of 320 internal medicine patients, exposure to a patient-centered, patient-informed educational video and handout based on the USDA-recommended MyPlate diet led to increased knowledge of the diet, the perception that making changes to diet would be easier, and a higher likelihood of talking to a physician regarding a healthy diet. These findings are in line with the literature and are important because we know that patients who receive dietary advice from their physicians, compared to patients who are simply handed the materials, are more likely to learn about and implement healthy behaviors.^15^

Furthermore, those who saw the video and received the handout enjoyed these materials overall, found them helpful, and felt motivated to change their diet because of them. This perception was likely enhanced by the cultural relevance and health literacy level of the materials, emphasizing the importance of patient education regarding healthy behaviors and the patients’ overall enjoyment of such interventions. As reported in other studies, our patients liked learning about healthy behavior via multiple media modalities.^13^

Based on the patients’ answers regarding their likelihood to discuss diet with their physician, we propose that this educational intervention could improve communication between patients and their physicians. Although patients stated that they were more likely to discuss diet with their physicians after exposure to the video, we only observed a trend toward statistical significance. However, with 30% of the primary care clinic population not exposed to the intervention, relationships between the educational intervention and improved patient-physician communication may be weaker than portrayed.

Another limitation of this study is that it was not randomized. Lack of randomization was because of the quality improvement nature of the intervention and ease of implementation of the intervention in 1 clinic. Ideally, patients would have been randomized, and more objective, long-term data such as food frequency and dietary habit questionnaires would have been measured to provide quantitative results of this educational intervention. Randomization of subjects would have accounted for unknown differences in characteristics between patient groups in different clinics and allowed for a better comparison. The subspecialty care group of patients likely had some inherent differences from the healthier primary care population that could have contributed to differences in the results. The design did not control for certain clinical conditions leading to more or less interest in dietary changes, such as patients with renal disease needing to adhere to a strict renal diet. However, the focus of this study was more on the feasibility of implementing the video and handout in the clinic and observing its effects and not on limiting variables to allow for a more controlled study. Ideally, 2 locations, 1 with the educational intervention and 1 without, could prevent crossover and allow for a better comparison between groups, although this design could potentially introduce other confounders.

Another limitation of the study is that the intervention continued throughout the study, allowing for the possibility of regurgitation of material vs true learning. Although we believe that most patients in the intervention group completed the survey based on seeing the educational video at a previous visit, patients may have kept the survey and completed it after seeing the video that day, reflecting immediate recall instead of learning.

The cost of creating the educational video was the majority of the budget for this study. The remaining costs included the cost of printing handouts and providing refreshments for nurses and physicians while educating them on the MyPlate diet and implementation of the study. Our hope is that this video can be used in a similar way in other clinics with like populations.

## CONCLUSION

This study suggests that using multiple modalities including handouts and a patient-centered video to educate patients about healthy diet is enjoyable to patients and effective in teaching, motivating change, and encouraging communication between patients and physicians.

## Notes

*This article meets the Accreditation Council for Graduate Medical Education and the American Board of Medical Specialties Maintenance of Certification competencies for Patient Care, Medical Knowledge, and Practice-Based Learning and Improvement.*
